# The Synergetic Effect of Plyometric Compound Exercises and Transcranial Direct Current Stimulation on Balance and Physical Function

**DOI:** 10.3390/healthcare11202774

**Published:** 2023-10-20

**Authors:** Jeong-Hyeok Song, Jong-Eun Yim

**Affiliations:** Department of Physical Therapy, The Graduate School of Sahmyook University, Seoul 01795, Republic of Korea; ssong2919@hanmail.net

**Keywords:** plyometric compound exercise, transcranial direct current stimulation, balance, power, agility

## Abstract

This study aimed to investigate the effects of plyometric compound exercises and Transcranial Direct Current Stimulation (tDCS) on balance and body function in healthy adults. Forty-five students enrolled at Noryangjin Y Academy in Seoul who met the research criteria were equally and randomly divided into the following groups: the Experimental Group I, Experimental Group II, and Control Group. Experimental Groups I and II received tDCS and sham tDCS for 20 min, respectively; both groups performed plyometric compound exercises for 30 min twice weekly for four weeks. The Control Group received sham tDCS for 20 min twice weekly for four weeks. Tests such as the static balance test (Functional Reach Test, FRT), dynamic balance test (Y-Balance Test, Y-BT), power test (vertical jump test and long jump test), and agility test (*t*-test and side-step test) were conducted on the day of the experiment, before and after the intervention. Experimental Groups I and II significantly improved in static balance, dynamic balance, power, and agility (*p* < 0.001), whereas the Control Group did not. Experimental Group I showed greater effects on static balance, dynamic balance, power, and agility than Experimental Group II and the Control Group (*p* < 0.001). In conclusion, plyometric compound exercises + tDCS intervention can be effective for an ordinary person who trains balance and body functions (power and agility); in particular, to improve exercise performance.

## 1. Introduction

Despite the positive effects of sports activities, exercise may have some negative consequences, the most prominent of which is injury during exercise [1]. Excellent balance, power, and agility are required to avoid injury during exercise and to achieve a high level of physical ability [2]. Therefore, balance control and improvement in physical function are the most important goals of exercise [3]. The relationship between balance, trunk control, and sports-related injury has been emphasized, and plyometric, agility, balance, and core stability exercises have been introduced as comprehensive injury-prevention training [4]. In the exercise training field, neuromuscular training programs (multi-intervention programs in which balance, muscular strength, plyometrics, agility, and exercise for each sport are combined), including balance training, are performed to optimize body function, prevent injuries, or rehabilitate the body [5]. 

The plyometric stretch-shortening drill exercises involve performing upper and lower extremity exercises using muscle load, reactive response, and functional movement patterns from eccentric contraction at a high speed to concentric contraction [6,7]. This exercise is characterized by eccentric contraction during the stretching of muscles and an immediate change in the contraction direction of the same muscle into concentric contraction; the shorter the amortization phase, which indicates a middle phase between the stretch and contraction, the more effective and powerful the performance of the exercises [7]. Plyometric exercise, which has various levels and is necessary for rehabilitation, can be performed across a spectrum ranging from low to high levels and helps to enhance the ability to control post-injury neuromuscular impairments and prepare for exercise capability [8]. Moreover, it has been reported that it is suitable for everyone, regardless of sex and age. It is effective in improving muscle strength, power, static and dynamic balance, and the prevention of injuries [9]. Additionally, when compared with balancing exercise, it showed a similar balancing improvement [9]. Moreover, it was recently demonstrated that plyometric and strength training exercises, including neuromuscular training components, as an injury prevention program are comprehensively effective in improving balance, agility, jumps, and speed [10]. 

The Transcranial Direct Current Stimulation (tDCS) is a stimulation method that alters neuronal excitement by stimulating the cerebral cortex through a microcurrent between an anode and cathode [11]. Noninvasive brain stimulation techniques, initially developed for clinical purposes, have expanded to the field of sports performance owing to the development of neuroscience in the 21st century; among them, tDCS is the most commonly used due to its safety, low cost, and ease of implementation [12]. Furthermore, tDCS is spotlighted due to its potential effects on brain activity by modulating neural excitement through noninvasive brain stimulation in nonathletes and athletes [13]. Additionally, it alters brain excitability in the long term after ceasing stimulation of a weak current (2–3 mA) directly to the scalp [14]. A study investigating tDCS efficiency in improving training outcomes, exercise performance, and muscular strength demonstrated a positive effect in 66.7% of the parameters tested [13]. It has been reported that the synergistic effect of tDCS and exercise not only improves exercise ability, postural control, and balance but also prevents falls [15]. 

A recent study reported that stimulating the primary motor area with tDCS to obtain neuromuscular advantages in athletes and nonathletes during exercise improves individual performance of exercises, increases the vertical height of jumping, amplitude of sit and reach, and endurance running capacity [16]. Additionally, because the tDCS equipment is convenient and wearable, tDCS can be a good method for nonathletes who want to enhance their strength in daily life or in terms of exercise abilities [16].

In summary, there is no prior study on an intervention of applying plyometric exercise and tDCS to both athletes and nonathletes. Furthermore, there are insufficient integrated studies on plyometric exercise and tDCS applied to athletes. Therefore, this study aims to find effects of the intervention of applying plyometric exercise and tDCS to nonathletes. Furthermore, there are insufficient integrated studies on plyometric exercise and tDCS. As the interaction between different exercise components may lead to synergistic effects, compound exercises are often performed in exercise-related intervention studies. Accordingly, the integration of plyometric compound exercises and tDCS, which have different exercise components, may have additional benefits rather than individual applications, as shown in previous research. Therefore, this study aimed to investigate the synergetic effects of plyometric compound exercises and tDCS on balance and physical function.

## 2. Materials and Methods

### 2.1. Participants

This study was conducted with 45 healthy adults in their 20s and 30s who were enrolled at the Y Academy in Noryangjin, Seoul, Korea. The participants understood the present study and consented to participation. They were classified into the following three groups: the Experimental Group I (*n* = 15; plyometric compound exercises + tDCS), Experimental Group II (*n* = 15; plyometric compound exercises + sham tDCS), and Control Group (*n* = 15; received sham tDCS). The results were analyzed using G*Power software (version 3.1.9.7) [17]. Based on the software power analysis (effect size = 0.5, significance level = 0.05, statistical power = 0.80), 42 participants were initially included, and 45 participants were finally included. Groups were created using randomization software (random allocation software program for windows 2.0, Isfahan University of Medical Sciences, Isfahan, Iran). At Y Academy located in Noryangjin, Seoul, the indoor temperature of the sports room was maintained at 25 degrees from 1 August 2023 to 15 September 2023. The intervention of the examinees was carried out in comfortable short-sleeved shirts. The experiment was conducted in a quiet room to minimize environmental distractions, and the environment was kept the same for the pre- and post-test.

The inclusion criteria were the following: regular adults who had not been engaged in regular athletic activities nor had any experience of plyometric exercise; those who were able to perform necessary tests and interventions; those who were sufficiently motivated; those who were able to perform an independent sports life and activities; those who were able to faithfully comply with the investigator’s controls; and those who provided consent to participate in the study. Candidates excluded from the study had the following features: a metal implant near the current-stimulated area, defective skull bones, history of epilepsy and seizures, psychiatric disorders, signs of depression, sensory impairment, vestibular disorders, post-traumatic stress disorder, skin wounds, or skin disease in the area to be measured, and a reason to be determined as inappropriate for the study. The study was conducted only with participants who understood the experiment; received a full explanation of the experimental procedure, process, and expected effects; and signed a written consent form. The study protocol was approved by the Institutional Review Board of Sahmyook University (SYU 2022-06-011-005).

### 2.2. Study Design

**Figure 1:**
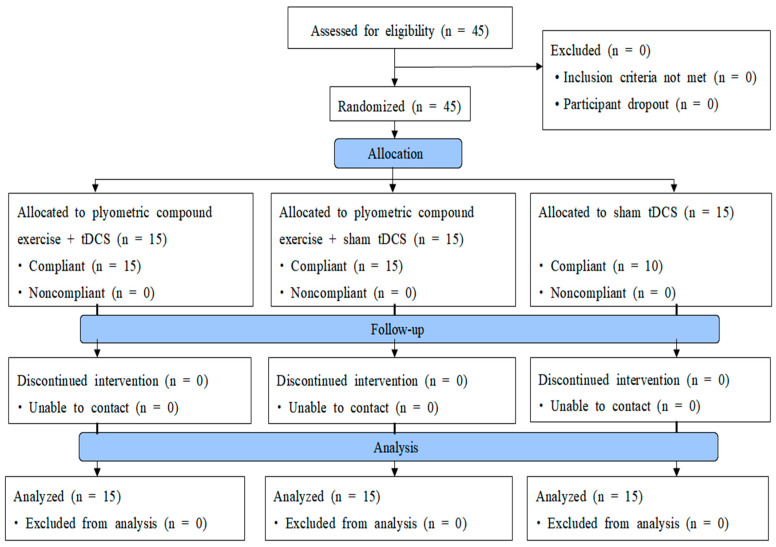
A flowchart of the study.

A three-group experimental design was used. The study compared pre-/post-test in each group and the difference between groups (Figure 1).

### 2.3. Procedures

The present study was conducted with 45 healthy adults in their 20s and 30s who were enrolled at the Y Academy in Noryangjin, Seoul, Korea. The 45 participants were randomly classified into the following three groups: the Experimental Group I (*n* = 15; plyometric compound exercises + tDCS), Experimental Group II (*n* = 15; plyometric compound exercises + sham tDCS), and Control Group (*n* = 15 participants; received sham tDCS). Tests such as the static balance test (Functional Reach Test, FRT), dynamic balance test (Y-Balance Test, Y-BT), power test (vertical jump test and long jump test), and agility test (*t*-test and side-step test) were conducted on the day of the experiment, before and after the intervention. After assessment before the intervention, the Experimental Group I received tDCS stimulation of the primary motor area for 20 min before performing the plyometric compound exercises. Experimental Group II received sham tDCS for 20 min, i.e., they performed the plyometric compound exercises without stimulation. The Control Group received sham tDCS for 20 min, i.e., did not receive any stimulation or perform the exercise. The assessments were conducted twice weekly for four weeks after the intervention.

### 2.4. Outcome Measures

#### 2.4.1. Static Balance Ability

An FRT was conducted to measure static balance; it was assessed as follows: the participant stood with feet shoulder-width apart on a fixed support surface; the upper arm joint was raised 90° to the front while the feet remained stationary; the participant kept their feet on the base of the support until they lost balance; the upper arm was stretched as far forward as possible; and the distance between the initial point at the end of the third carpometacarpal joint in the starting position and the final point at maximum forward extension was measured. If the measurement was ≤15–17.5 cm, it was concluded that the functional balance was limited [18]. Additional attention was paid to avoid posterior hip dislocation and knee flexion, which reduce the reliability and validity of the FRT assessment. Distance measurements were performed using a tape measure attached to a wall. In a previous study, the test–retest reliability of the FRT in 128 participants aged 20–80 years was r = 0.92, and the inter-rater reliability was r = 0.98, showing high reliability [18]. For data collection, the maximum distance reached during three repeated measurements was recorded to minimize measurement error.

#### 2.4.2. Dynamic Balancing Ability

To measure dynamic balance ability, the Lower Quarter Y-Balance Test (YBT-LQ) was used with a Y-BT tool (Y-BT Kit; Functional Movement Systems, Inc., Chatham, MA, USA). The Y-BT tool, which is an evolved tool of a very high reliability test—the modified Star Excursion Balance Test (mSEBT) (ICC = 0.91)—is a simple and reliable test that assesses dynamic balance in three directions: anterior, posterior medial, and posterior lateral [19,20]. Before the measurement, the participants were thoroughly familiarized with the Y-BT. Subsequently, they practiced the test three times, using their arms and legs to push the box mounted on the tool in three directions as much as possible. In the actual measurement, the participants were asked to push the box three times in each of the three directions; the maximum distance (cm) reached was recorded [19]. The measurements in the three directions were summed to consider the effect of leg length on the test and then standardized by dividing the sum by three times the leg length and multiplying by 100 to consider the deviations caused by height [21]. To standardize, after the participant lay down in an anatomical posture on the floor, the leg length (cm) was measured from the anterior superior iliac spine to the medial malleus. The YBT showed high intrarater (ICC 0.85–0.91) and interrater (ICC 0.99–1.00) reliabilities [19].

#### 2.4.3. Power

1. The vertical jump test: The participant stood 20 cm away from a wall with feet shoulder-width apart, outstretched arms as far as possible, marked the height with fingertips, jumped as high as possible without run-up, and marked the points of fingertips as the height; the distance between the first and second marks was measured in cm. The entire process was repeated twice, and the highest measurement was recorded [22].
2. The long jump test: The participant stood in front of a parallel measuring board, lowered their center of body weight, bent their knees, swung their arms backward, jumped off the ground, swung their arms forward as much as possible, stretched their body, bent their abdomen and legs, and landed on the heels; the researcher measured the distance between the heels when landing on both feet on the measuring board. The entire process was repeated twice, and the highest measurement (cm) was recorded [23].

#### 2.4.4. Agility

1. *t*-test: A stopwatch (CASIO HS-80W, Japan) was used to measure agility. The participant quickly sprinted from point A to point B (9.14 m) following the instruction “Ready, start”. The participant then moved their body to the right to point D (4.57 m), from point D to point C, and back to point B (13.71 m), before finally returning to point A (9.14 m). The entire process was repeated twice, and the shortest time(s) was recorded [24].
2. Side-step test: Three lines were marked with paper tape at the center, on the left, and on the right at an interval of 120 cm on the floor. The participant took a ready posture with both feet shoulder-width apart; as the test began, if the right foot crossed over to the right line, it was scored as 1; if the right foot, which had crossed over to the right line, quickly returned to the center line, it was 2; if the left foot crossed the left line after returning to the center line, it was 3. The whole process was repeated twice for 10 s and the maximum number of frequencies was recorded [25,26].

### 2.5. Intervention

#### 2.5.1. Exercise Program

For this experiment, four types of exercise programs were performed twice a week for four weeks, with three–four sets for each exercise program, and the number of sets was gradually increased. In the literature, plyometric and tDCS studies have been conducted for as short as 4 weeks and as long as 8 weeks. However, due to the fact that we were conducting the experiment during the COVID-19 pandemic, we decided to run it for 4 weeks. However, we believe that four weeks is a reasonable amount of time to see some benefit (see limitations section). In this study, a 30 min plyometric exercise program was performed twice a week for four weeks. It was divided into 5 min of warm-up, 20 min of main exercise, and 5 min of cool-down exercise. It has been reported that plyometric exercise requires a sufficient break time between sets as it involves anaerobic activities requiring maximum effort [27]; thus, this program provided a break of 2 min between sets.

To properly perform the plyometric exercise, the participants were classified into three groups according to the number of times their feet were in contact with the ground: 80–100, beginner; 100–120, intermediate; and 120–140, advanced [28]. In this study, the number of ground contacts was limited to 110–130.

For box exercises, the National Strength and Conditioning Association (NSCA) recommends a box height of 15–107 cm; however, the present study used a box with a height of 51 cm to prevent injury to beginners. Verbal instructions and supervision were provided to minimize the contact time of the ground reaction forces during the jump and maximize the height of each jump [29]. Before the experiment, two orientation sessions were held with the participants, and a warm-up exercise was performed with a thorough understanding of the experiment’s purpose. The posture and precautions were explained to the inexperienced participants, who sufficiently practiced the postures beforehand. The program was revised and adapted from a previously described program [30] mentioned in the NSCA [28] (Table 1).

#### 2.5.2. Transcranial Direct Current Stimulation

tDCS is a noninvasive technique that changes the excitability of neurons in localized areas of the brain by applying a weak current through the scalp. In this study, a tDCS device (Halo Sports 2, Apsun, Inc., Republic of Korea) approved by the U.S. Food and Drug Administration (FDA) was used to stimulate the primary motor area. The participants were instructed to wet the rubber pad attached to the headset with water and wear it to allow the entire electrode to be close to the scalp. In addition, the headset was fixed in consideration of the intervention characteristics of the three groups, and the participants were instructed to request the cessation of the experiment if they experienced any inconveniences. Furthermore, they were informed in advance about the possibility of an uncomfortable feeling even with a very small current flow. The Experimental Group I (plyometric compound exercises + tDCS) received tDCS for 20 min, following which they performed the plyometric compound exercises. The Experimental Group II (plyometric compound exercises + sham tDCS) received a recognizable tDCS for 30 s in order to identify the sham effect. Subsequently, the stimulation was ceased, and the participants received the sham stimulation for 20 min, following which they performed the plyometric compound exercises [31]. The Control Group received recognizable stimulation for 30 s, in the same way as stimulating the experimental groups, using an efficient blinding method demonstrated by prior studies. The stimulation was then ceased, and then the participants received the sham stimulation for 20 min [31]. The experiment was conducted in a quiet room to minimize environmental distractions, and the environment was unchanged before and after the intervention. The intensity of the stimulation, which was repeated twice a week for four weeks, was kept below 2–3 mA and the duration was 20 min with an additional 5 min of relaxation time for safety and effectiveness based on previous studies [32,33].

#### 2.5.3. Statistical Analysis

All results and statistical analyses were processed using SPSS 23.0. All participants were tested for normality with satisfactory results. General characteristics of the participants were determined using frequency analysis and descriptive statistics. Balance, power, and agility within the group before and after the intervention were compared using paired *t*-tests. To determine the group effect on outcome measures, 2 × 3 (Time by Group) repeated measures ANOVAs were performed with Time (pre-test and post-test) as the repeated factor and Group as the between-subjects factor. When statistical significance was reached, post hoc tests were performed using Tukey’s hostility significance method; the significance level was set at *p* < 0.05.

## 3. Results

### 3.1. General Subject Characteristics

No significant differences between groups were found for baseline demographic characteristics (*p* > 0.05) (Table 2).

### 3.2. Changes in Static Balance (FRT) before and after the Intervention

Experimental Groups I and II showed significant improvements in static balance within the group (*p* < 0.001); however, the Control Group showed an insignificant improvement. There were significant differences in the group-by-time interaction for the FRT (*p* < 0.001) (Table 3).

### 3.3. Changes in Dynamic Balance (Y-BT) before and after the Intervention

#### 3.3.1. Change in Dynamic Balance of the Left Leg before and after the Intervention

Experimental Groups I and II showed significant improvements in the dynamic balance of the left leg within the group (*p* < 0.001); in contrast, the Control Group showed an insignificant improvement. There were significant differences in the group-by-time interaction for the dynamic balance of the left leg (*p* < 0.001) (Table 4).

#### 3.3.2. Change in Dynamic Balance of the Right Leg before and after the Intervention

Experimental Groups I and II showed significant improvements in the dynamic balance of the right leg within the group (*p* < 0.001); in contrast, the Control Group showed an insignificant improvement. There were significant differences in the group-by-time interaction for the dynamic balance of the right leg (*p* < 0.001) (Table 4).

### 3.4. Changes in Power before and after the Intervention

#### 3.4.1. Vertical Jump Test

Experimental Groups I and II showed significant improvements in the vertical jump height within the group (*p* < 0.001); in contrast, the Control Group showed an insignificant improvement. There were significant differences in the group-by-time interaction for the vertical jump test (*p* < 0.001) (Table 5).

#### 3.4.2. Long Jump Test

Experimental Groups I and II showed significant improvements in the long jump distance within the group (*p* < 0.001); however, the Control Group showed an insignificant improvement. There were significant differences in the group-by-time interaction for the long jump test (*p* < 0.001) (Table 5).

### 3.5. Changes in Agility before and after the Intervention

#### 3.5.1. *t*-Test

Experimental Groups I and II showed significant improvements in agility within the group (*p* < 0.001); however, the Control Group showed an insignificant increase. There were significant differences in the group-by-time interaction for the *t*-test (*p* < 0.001) (Table 6).

#### 3.5.2. Side-Step Test

Experimental Groups I and II showed significant improvements within the group in the side-step test (*p* < 0.001); however, the Control Group showed an insignificant improvement. There were significant differences in the group-by-time interaction for the side-step test (*p* < 0.001) (Table 6).

## 4. Discussion

In this study, regarding the change in static balance before and after the intervention, Experimental Groups I and II showed significant improvements within the group (*p* < 0.001). The Control Group also showed no improvement. In addition, there were significant differences in the group-by-time interaction for the static balance (*p* < 0.001).

Regarding the changes in the dynamic balance of the left and right legs before and after the intervention, Experimental Groups I and II showed significant improvements within the group (*p* < 0.001). However, the Control Group also showed no improvement. In addition, there were significant differences in the group-by-time interaction for the dynamic balance of the left and right legs (*p* < 0.001). These results, similar to those of previous studies, suggest that plyometric exercise, a type of neuromuscular exercise, increases joint stability and improves neuromuscular coordination, thereby increasing functional joint stability [34,35]. In particular, plyometric exercises involving jumping and landing movements have been reported to be effective in improving balance by increasing the proprioceptive sense of the ankle joint during weight shifts [36]. In line with the present study, a previous study reported that female badminton players aged 15–25 years improved their balance ability by performing plyometric exercises three times a week for six weeks, as measured by the YBT-LQ [37]. On the contrary, a study on tDCS reported that anodal tDCS applied to the primary motor area of healthy young adults for 10 min induced an increase in corticospinal excitation, thus improving static postural stability while standing quietly with the eyes closed [38]. This application could improve the dynamic balance in healthy young adults [39]. In congruence with a prior study, the improved balance performance was attributed to an increase in neurotransmission velocity, motor performance, and muscle strength due to the stimulation of the primary motor area [13]. The increase in muscle strength may have reduced the abnormal muscle tone, thereby facilitating movement control and reducing postural sway, which improved dynamic balance [40]. In addition, it can be inferred that the improved proprioceptive sense, i.e., the sense of joint position, had a positive effect on balance ability [41]. Controlling cortical excitability increases concentric signal sensitivity, and thus, an increased somatosensory stimulation evokes potential and cerebral blood flow improves balance through proprioceptive sensation [42,43,44].

Regarding the vertical jump test of power before and after the intervention in the present study, Experimental Groups I and II showed a significant improvement within the group (*p* < 0.001), while the Control Group showed no improvement. In addition, there were significant differences in the group-by-time interaction for the vertical jump test (*p* < 0.001).

In addition, regarding the change in the long jump test before and after the intervention, Experimental Groups I and II showed a significant improvement within the group (*p* < 0.001), while the Control Group showed no improvement. In addition, there were significant differences in the group-by-time interaction for the long jump test (*p* < 0.001).

Similar to a previous study, these significant results show that plyometric exercises enhance the proprioceptive sense and improve neuromuscular control through the stimulation of nerve receptors around the muscle spindle, Golgi tendon organ, joint capsules, and ligaments [45]. In addition, plyometric exercises are deemed to activate the sensitivity of the nervous system by utilizing the elastic components of muscles and tendons and the stretch reflex through stretch-shortening drills, and they increase the responsiveness of the musculoskeletal system, resulting in an increase in power [46]. Similar to the present study, a prior study that compared strength, jumping, and functional abilities of healthy elderly people who were classified into three groups (experimental group I, who performed the plyometric exercise; experimental group II, who performed resistance exercise only; and experimental group III, who performed the conventional walking exercise three times a week for 12 weeks) reported that the experimental group I showed a greater effect than the other two groups [47]. In contrast, 13 basketball players received tDCS with a current of 2 mA to the primary motor area for 20 min prior to sprinting. The results showed that the intervention did not affect heart rate or perceived athletic grade but prevented fatigue and speed reduction during sprinting and dynamic jump performance [48]. Thus, it can be concluded that primary motor area stimulation through tDCS improves balance, strength, and jumping ability, thereby enhancing physical function and exercise performance.

Regarding the t-test changes in agility before and after the intervention in the present study, Experimental Groups I and II showed a significant reduction within the group (*p* < 0.001), while the Control Group showed no improvement. Furthermore, there were significant differences in the group-by-time interaction for the *t*-test (*p* < 0.001).

In addition, regarding the change in the side-step test before and after the intervention, Experimental Groups I and II showed a significant improvement within the group (*p* < 0.001), while the Control Group showed no improvement. There were significant differences in the group-by-time interaction for the side-step test (*p* < 0.001).

These findings suggest that plyometric exercises using stretch-shortening drills improve muscle strength due to the repetitive contraction movements of large muscles in accordance with the phenomena of extension reflexes, muscle elasticity, and adaptation of the muscular nerves to the Golgi tendon organ. Plyometric exercises promote the sensory and motor functions of the muscular nerves by stretching the ligaments and tendons connected to the joints, thereby developing body control and coordination, and improving agility [46,49]. Consistent with the present study, a prior study reported that agility improved more in the experimental group who performed plyometric + dynamic stability compound exercises twice a week for 30 min for six weeks than in the active control group [50].

In a study on tDCS in boxers whose accuracy, agility, and endurance are important, it was shown that improvements in selective attention, reaction time, and motor performance were seen when the primary motor and supraspinal (hand) regions were simultaneously stimulated with tDCS, a noninvasive central nervous system stimulation technique [51]. This indicates that tDCS of the primary motor area plays an important role in increasing corticospinal excitability and reaction speed [52]. Similar to prior studies, the improvement in agility stems from the tDCS-induced changes in cortical excitability. Subsequently, this improved agility increases the proprioceptive sensibility of specific concentric signals occurring from joints, ligaments, and tendons, thereby increasing the neuromuscular excitability and reactivity to improve the performance of physical functions [7,36].

Many studies, including the present study, have shown that plyometric exercises are suitable for people of all ages and effective in improving strength, power, injury prevention, and static and dynamic balances [9]. In addition, tDCS combined with exercise has been shown to improve balance and physical performance [53] and can be suggested as a good approach to improve muscle strength and physical functions in ordinary people [13]. According to the results of the present study, plyometric compound exercises and tDCS, which can be utilized by ordinary people in the midst of their busy lives due to the short exercise and stimulation time, significantly impacted balance and physical function. Additionally, the stimulation of the primary motor area by pure tDCS was effective.

The limitations of the present study are as follows: The number of participants was limited. The recruitment and measurements were conducted in one location; thus, the present study cannot generalize its conclusion to everyone. The duration of the intervention (four weeks) was short; thus, the ability to determine the long-term effects of the intervention based on the results of the present study are restricted. In the literature, plyometric and tDCS studies have been conducted for as short as 4 weeks and as long as 8 weeks. However, due to the fact that we were conducting the experiment during the COVID-19 pandemic, we decided to run it for 4 weeks. However, we believe that four weeks is a reasonable amount of time to see some benefit. Considering these limitations, future studies should recruit a larger number of participants than those in the present study and apply the intervention continuously with variable application times.

## 5. Conclusions

This study investigated the synergetic effects of plyometric compound exercises and tDCS on balance, power, and agility in 45 healthy adults and there were significant differences in the group-by-time interactions for the balance, power, and agility (*p* < 0.001).

The present study suggests that plyometric compound exercises and tDCS can be effective interventions to improve balance, power, and agility in ordinary adults who aim to improve their quality of life and athletic performance and that the application of plyometric compound exercises and tDCS is more effective than plyometric exercise alone. Future studies based on different dependent variables, intervention methods, and a wider range of ages and participants are necessary to provide more clinical evidence.

## Figures and Tables

**Table 1:** Four exercise programs of the study.

	Type of Exercise	Reps	Sets
Week 1	Squat Jump, Side Short Pitch, Lateral Barrier Hop, Skip	10	3
Week 2	Squat Jump, Two Foot Ankle Hop, Ice Skater Jump, Lunge Hop	10	3
Week 3	Two Foot Ankle Hop, Ice Skater Jump, Squat Box Jump, Lunge Hop	12	3
Week 4	Two Foot Ankle Hop, Squat Box Jump, Burpee Box Jump, Leap Frogs Jump	15	4

**Table 2:** General subject characteristics.

	Experimental Group I (*n* = 15)	Experimental Group II (*n* = 15)	Control Group (*n* = 15)	*p*
Age (years)	33.06 ± 4.60 ^a^	33.26 ± 4.80	33.00 ± 3.22	0.984
Height (cm)	174.60 ± 2.82	174.8 ± 3.27	176.4 ± 4.32	0.293
Weight (kg)	75.40 ± 3.85	74.86 ± 4.06	75.66 ± 4.59	0.868

^a^ Mean ± standard deviation.

**Table 3:** Changes in static balance (Functional Reach Test (FRT)) before and after the intervention (*n* = 45).

		Experimental Group I (*n* = 15)	Experimental Group II (*n* = 15)	Control Group (*n* = 15)	Group X Time F (*p*)
FRT (cm)	Before	42.00 ± 2.13 ^a^	41.86 ± 2.32	41.86 ± 2.77	46.214 (<0.001)
	After	46.66 ± 2.58	44.40 ± 1.91	42.06 ± 2.60	
	Difference	4.66 ± 1.49	2.53 ± 1.18	0.26 ± 1.03	
	t(*p*)	−12.081 (0.000)	−8.264 (0.000)	−0.823 (0.424)	

Note. FRT = Functional Reach Test (static balance test). ^a^ Mean ± standard deviation.

**Table 4:** Changes in dynamic balance (Y-Balance Test, Y-BT) before and after the intervention (*n* = 45).

		Experimental Group I (*n* = 15)	Experimental Group II (*n* = 15)	Control Group (*n* = 15)	Group X Time
					F (*p*)
Y-Balance Test (Rt score)	Before	90.83 ± 1.77 ^a^	91.37 ± 3.04	92.02 ± 2.29	55.131 (<0.001)
	After	94.25 ± 1.93	93.75 ± 2.83	92.03 ± 2.42	
	Difference	3.42 ± 1.17	2.38 ± 0.77	0.00 ± 0.72	
	t (*p*)	−11.290 (0.000)	−12.003 (0.000)	−0.045 (0.965)	
Y-Balance Test (Lt score)	Before	91.62 ± 1.41 ^a^	91.42 ± 2.58	91.95 ± 2.35	85.703 (<0.001)
	After	95.03 ± 1.49	94.09 ± 2.71	92.01 ± 2.32	
	Difference	3.40 ± 0.52	2.67 ± 0.90	0.06 ± 0.73	
	t (*p*)	−25.197 (0.000)	−11.475 (0.000)	−0.321 (0.753)	

Note. Y-Balance Test (right and left leg dynamic balance test). ^a^ Mean ± standard deviation.

**Table 5:** Change in results of the vertical and long jump test before and after the intervention (*n* = 45).

		Experimental Group I (*n* = 15)	Experimental Group II (*n* = 15)	Control Group (*n* = 15)	Group X Time
					F (*p*)
Vertical Jump Test (cm)	Before	40.20 ± 2.00 ^a^	40.46 ± 1.76	40.26 ± 2.25	57.512 (<0.001)
	After	45.20 ± 1.97	42.93 ± 2.84	40.33 ± 2.09	
	Difference	5.00 ± 1.25	2.46 ± 1.59	0.03 ± 0.79	
	t (*p*)	−15.448 (0.000)	−5.980 (0.000)	−0.323 (0.751)	
Long Jump Test (cm)	Before	229.60 ± 4.59 ^a^	230.13 ± 3.94	230.73 ± 4.62	107.043 (<0.001)
	After	235.00 ± 4.47	234.20 ± 4.00	230.93 ± 4.72	
	Difference	5.40 ± 1.18	4.06 ± 0.88	0.20 ± 0.94	
	t (*p*)	−17.676 (0.000)	−17.823 (0.000)	−0.823 (0.424)	

Note. Vertical Jump Test (vertical jump test), Long Jump Test (standing long jump test). ^a^ Mean ± standard deviation.

**Table 6:** Changes in the results of t-test and side-step test before and after the intervention (*n* = 45).

		Experimental Group I (*n* = 15)	Experimental Group II (*n* = 15)	Control Group (*n* = 15)	Group X Time
					F (*p*)
*t*-Test (second)	Before	13.22 ± 0.40 ^a^	13.16 ± 0.47	13.30 ± 0.38	35.942 (<0.001)
	After	12.17 ± 0.40	12.47 ± 0.56	13.32 ± 0.52	
	Difference	−1.05 ± 0.31	−0.68 ± 0.35	0.01 ± 0.37	
	t (*p*)	12.926 (0.000)	7.502 (0.000)	−0.171 (0.867)	
Side-Step Test (frequency)	Before	12.06 ± 1.27 ^a^	12.00 ± 1.30	10.86 ± 2.13	73.082 (<0.001)
	After	17.26 ± 2.15	15.73 ± 1.57	10.93 ± 2.12	
	Difference	5.20 ± 1.42	3.73 ± 1.38	0.06 ± 0.59	
	t (*p*)	−14.140 (0.000)	−10.425 (0.000)	−0.435 (0.670)	

Note. *t*-Test (*t*-type agility test), Side-Step Test (Side-Step Test). ^a^ Mean ± standard deviation.

## Data Availability

Not applicable.
